# Supplementary material for: Multicomponent dynamics in amorphous ice studied using X-ray photon correlation spectroscopy at elevated pressure and cryogenic temperatures
Source: Commun Chem. 2025 Mar 16;8:82. doi: 10.1038/s42004-025-01480-8 (PMC11911450; doi:10.1038/s42004-025-01480-8)
Supplement: Supplementary file 2 — Supplementary Information [file 42004_2025_1480_MOESM2_ESM.pdf]

Supplementary information

Multicomponent dynamics in amorphous ice studied using X-ray photon correlation spectroscopy at elevated pressure and cryogenic temperatures

Aigerim Karina[1,*], Hailong Li[2,3,*], Tobias Eklund[2,4,5], Marjorie Ladd-Parada[6], Bernhard Massani[7], Mariia Filianina[1], Neha Kondedan[1], Andreas Rydh[1], Klara Holl[2,4], Ryan Trevorah[8], Simo Huotari[8], Robert P.C. Bauer[9,10], Claudia Goy[9], Nele N. Striker[9], Francesco Dallari[9,+], Fabian Westermeier[9], Michael Sprung[9], Felix Lehmkühler[9,11], Katrin Amann-Winkel[2,4,1,#]

[1]Department of Physics, Stockholm University, Sweden
[2]Max-Planck-Institute for Polymer Research, Germany
[3]State Key Laboratory of Fine Chemicals, School of Chemical Engineering, Dalian University of Technology, China
[4]Institute of Physics, Johannes Gutenberg University Mainz, Germany
[5]European X-ray Free-Electron Laser, Germany
[6]Department of Chemistry, Glycoscience Division, KTH, Stockholm, Sweden
[7]The University of Edinburgh, School of Physics and Astronomy (SoPA), Centre for Science at Extreme Conditions (CSEC), Edinburgh, UK
[8]Department of Physics, University of Helsinki, Finland
[9]Deutsches Elektronen-Synchrotron DESY, Germany
[10]Freiberg Center for Water Research, Technische Universität Bergakademie Freiberg, Germany
[11]Hamburg Centre for Ultrafast Imaging, Germany

#Corresponding author. Email: amannk@mpip-mainz.mpg.de
*These authors equally contribute to this work.
+ Currently at: Department of Physics and Astronomy, University of Padova,

Introduction

The pressure dependence of the glass transition provides insight to relaxation dynamics and related viscosity changes at elevated pressure. This is of particular interest for the glassy states of water, where two amorphous states of different density exist. We present novel X-ray photon correlation spectroscopy (XPCS) experiments at elevated pressure and cryogenic temperatures using a diamond anvil cell (DAC). Extended fit procedures and additional data are summarized as follows.

Samples

We have measured in total three equilibrated high-density amorphous ice samples (eHDA). All samples have been prepared ex-situ using a piston cylinder setup, following a well-established protocol (see Methods and Materials of the main manuscript). [1], [2] The quench recovered samples have been transported in liquid nitrogen to the beamline. Prior to the experiment, the samples were cold-loaded into a diamond anvil cell (DAC), and carefully repressurized using the four screws on the DAC. The pressure was determined using ruby fluorescence at the lowest temperature. In Fig. 1C of the main manuscript the ruby measurement for sample A and B are compared. For sample B, main sample of the study, we measured the ruby spectrum three times, as shown in Fig. S1A. Spectra were fitted with a Gaussian function (scipy curve fit) to determine the peak maximum with an error of 0.003-0.004 nm. This corresponds to a pressure range of 0.07-0.1 Gpa for sample B (Fig. S1B),

centered around a mean peak position of 693.366 nm, equivalent to 0.08 GPa at 93 K. Ruby reference of the Ruby sphere was measured at ambient pressure at 296 K to be 694.21 nm.

The samples have been heated in-situ until crystallization or transformation to LDA. The three conditions are summarized in Table S1. The transition temperatures and related pressures, derived from the WAXS analysis (Fig. S2), are summarized in the phase diagram in Fig. 7 of the main manuscript.

Sample	Absorber	Photon flux density photons/(s μm^2)	Pressure (GPa) at 93 K
eHDA_A	12	1.3 x 10^7	0.08 $\pm$ 0.02
eHDA_B	18	6.5 x 10^6	0.08 $\pm$ 0.02
eHDA_D	18	6.5 x 10^6	0.75 $\pm$ 0.02

Table S1: Summary of eHDA samples measured by XPCS inside a DAC. "Absorber" describes the number of 25 μm thick silicon foils used as attenuator to mitigate radiation damage. 12 and 18 foils correspond to an X-ray transmission of 0.27 and 0.14 at 12 keV photon energy, respectively. The analysis of the correlation functions at the highest pressure was not possible, due to the appearance of Pseudo-Kossel lines (see section "Experimental setup").

During heating inside the DAC the P-T pathway will not be isobaric. The pressure is expected to decrease during heating. We relate to the initial pressure throughout the manuscript (Table S1), but tried to estimate the change as follows. We did independent measurements after the beamtime, both on the pressure evolution as well as on the sample temperature (see below). Fig. S1C shows three datapoints for low-density amorphous ice cold-loaded to the DAC and the related pressure change upon heating. The pressure decreases by ~3.5% by 10 K. In addition, we did the same test loading glycerol to the DAC in two different pressure ranges, and observed a percentual pressure change of 6-8% pressure decrease per 10 K step. In-situ pressure determination during the XPCS experiments was not possible for technical reasons. Using an average value, the pressure during our experiment might decrease at 123 K down to 0.071 GPa, representing a 11% pressure reduction during our measurements. This lies within the range of 0.08 $\pm$ 0.02 GPa.

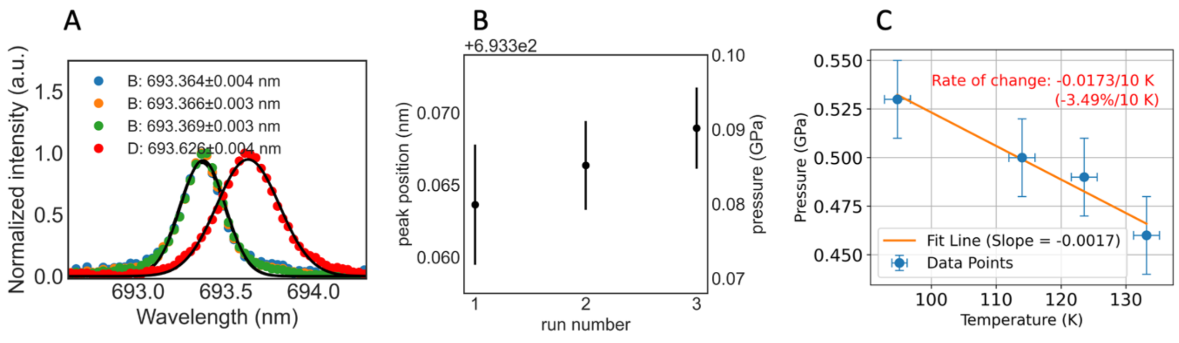

Fig. S1: (A) shows Ruby R1 lines of three independent Ruby measurements during the beamtime of sample B as well as on sample D (red). (B) shows the related peak position of sample B and the derived pressure, showing the uncertainty of the pressure determination. (C) shows an external measurement (after the beamtime) of cold-loaded LDA, to determine the pressure change upon heating.

Additional evidence for the sample pressure is the location of the first broad diffraction maximum as well as the observation to which phase the eHDA sample transforms to upon heating. [3], [4] The shift of the Q_{max} of eHDA upon pressure is well known from literature. [5] Additionally, a density and d-spacing relation was reported on quench recovered samples, recovered from different pressures. [6] We here observe a shift from Q = 2.03 $Å^{-1}$ (sample A/B) to Q = 2.17 $Å^{-1}$ (sample D). This clearly shows an increase in pressure, as always the same initial sample was loaded to the DAC and subsequently compressed. The position Q_{max} of sample D is in very good agreement with a pressure of 0.75 GPa, as determined by the Ruby setup. The pattern of samples A/B are almost identical to the quench recovered samples prior loading. While samples A and B transform to LDA(L) before crystallization, indicating a pressure < 0.1 GPa, sample D directly transforms to ice IX, indicating a pressure > 0.1 GPa.

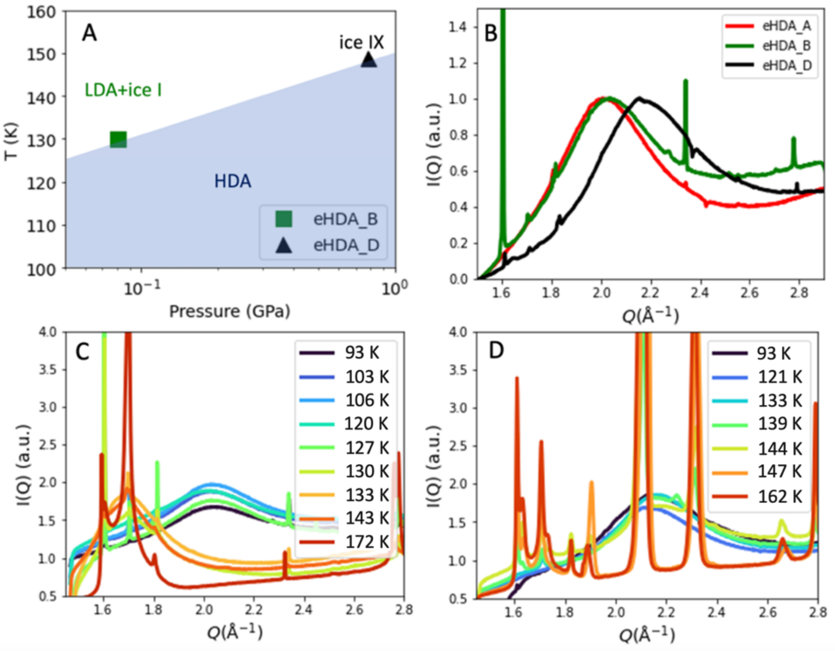

Fig. S2: (A) Phase diagram depicting the sample temperature and pressure, at which the different samples transformed to LDA, ice Ih and ice IX respectively. (B) WAXS I(Q) pattern for sample A, B, and D. The first diffraction maximum of eHDA at 93 K for sample D is clearly distinct (Q_{max} = 2.17 $Å^{-1}$) due to the higher pressure (0.75 GPa). This sample directly crystallizes to ice IX at (T_{sample} = 149 K). (C) WAXS I(Q) for sample B during heating. (D) WAXS I(Q) for sample D during heating.

Experimental setup and temperature calibration

A picture of our experimental setup is shown in Fig. S3a. The setup consists of a cubic vacuum chamber, a cryostat and a ruby fluorescence setup. Details are summarized in the main manuscript in the Methods section, additional information is provided here. To measure the pressure of the sample inside the DAC while mounted to the beamline (Fig. S3A), we used a rotational flange (VAb DDF 100P) between the cold finger and the vacuum chamber. This allowed us to determine the pressure before and after the XPCS experiments by rotating the cold finger 90 degree and making the DAC facing the ruby fluorescence setup (Fig. S3A, number 5).

DAC was clamped into a two-part Cu block via screws (Fig. S3B). Since there remains a small gap between Cu-block and DAC, in particular during cold-loading procedures, the thermal contact between copper holder and DAC is not perfect. This leads to a temperature offset between the measured temperature during the experiment and the sample compartment inside the DAC (T_{sample}). During the experiments, the temperature at the bottom of the cryostat (JANIS ST-400) was measured using the inbuilt Si-diode (sensor A) as well as an additional Si-diode (sensor B, as visible in Fig. S3B_1). During heating, sensor A and B show a small thermal lag, sensor B follows sensor A with some delay, but stabilizes after 20 min. Figure S4 and Table S2 show the temperature protocol of samples A and B. The sample temperature inside the

DAC must have an even larger offset. After the X-ray experiments, we calibrated this offset by gluing a thermocouple (type K) onto the gasket inside the DAC. The results for the cooling are shown in Fig. S3-D, data during step-wise heating with 30 min annealing time are shown in Fig. S4-A.

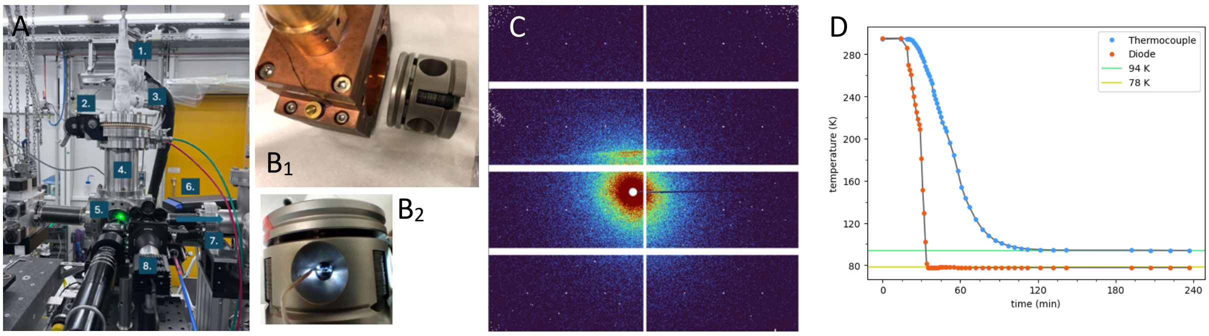

Fig. S3: A) Customized experimental setup at beamline P10 (PETRA III, DESY). 1. Cryostat, 2. Rotational Flange, 3. Temperature cable, 4. Vacuum chamber, 5. Microscope, 6. WAXS Detector, 7. Towards SAXS detector, 8. Laser System B) Cold-head with Cu-sample holder and DAC, B_1 as used during the beamtime, B_2 with glued thermocouple. C) SAXS detector image of sample D with strong Pseudo-Kossel line, or another scattering artefact. D) External temperature measurement during cooling to quantify the offset between cryostat finger and sample compartment. Heating related data see Fig. S4.

The temperature during the experiment was changed at a rate of 5 K/min. The Inbuilt Si-diode (sensor A), as purchased from JANIS, exactly follows at this rate, we did not observe any overshoot effect, once the set-temperature is reached the given value oscillates by $\pm$ 0.1 K (slightly more at lower temperatures). We changed the temperature in steps of 10 K at low temperature and 3-4 K at the higher temperatures. The exact protocol is graphically shown in Fig S5. At each temperature step we measured three to four XPCS series (1000s each) at different sample spots, the series discussed within the paper are marked coloured. The first series was started directly when sensor A reached the set temperature, and is affected by equilibration. This is, it usually shows faster and heterogeneous dynamics, therefore only the series measured after the equilibration time of 20 min (1000s + time to find a new sample spot) or at later times is used for the analysis. Searching a new spot and changing temperature is done by hand and not fully automated. Fig. S4-A shows an external measurement for diode A and a thermocouple glued to the gasket (see above). The plot shows that the sample temperature (thermocouple) lags behind but reaches a plateau after 20-30 min, which is when we started the XPCS experiment. In total we did four external heating runs, each with a freshly glued thermocouple, the averaged data are summarized in Figure S4-B. We estimate the temperature offset during the XPCS experiment using the equation shown in Fig. S4-B, the values are provided in Table S2.

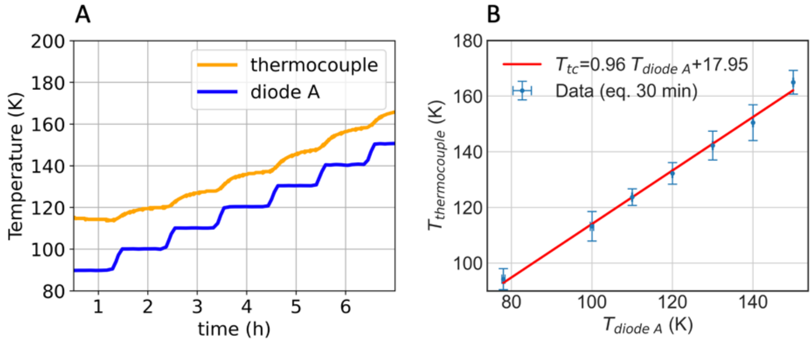

Fig. S4: External measurement with thermocouple (TC) glued to gasket, heating in steps of 10 K and equilibrated for 30 min. B shows the offset between thermocouple and Si-diode A after 30 min. The determined function can be used to estimate the sample temperature during the XPCS experiments.

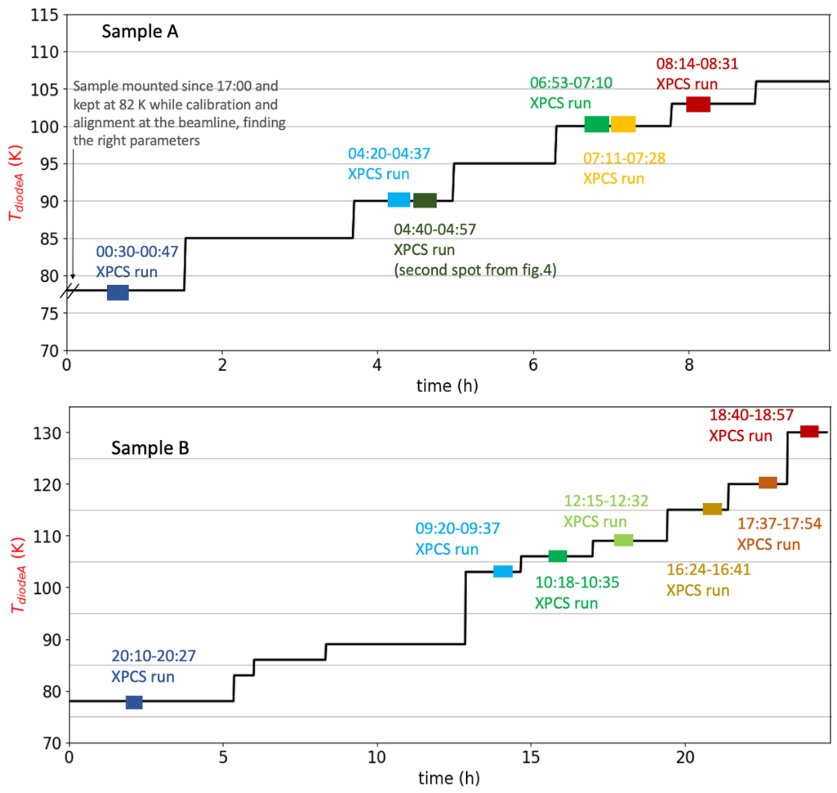

Fig. S5: Temperature protocol for samples A and B. XPCS data shown in the main manuscript are marked colored and include the timestamp from during the experiment.

During beamtime			T_{sample}
Diode A = set Temp. heating	Diode B after 20-40 min equilibration		after equilibr. (calculated from equation in Fig. S4)
Sample A			
77.5 +- 0.4	82 +- 0.4		93
85 +- 0.3	93.2 +- 0.3		
90 +- 0.4	103.8 +- 0.4		104
95 +- 0.3	108		
100 +- 0.3	110.7 +- 0.3		114
103 +- 0.2	112.5 +- 0.2		117
106 +- 0.1	115 +- 0.1		120
Sample B			
77.5 +- 0.4	81.9 +- 0.4		93
83 +- 0.3	93.7 +- 0.3		
86 +- 0.4	98 +- 0.4		
89 +- 0.4	103 +- 0.2		
103 +- 0.1	108.7 +- 0.1		117
106 +- 0.1	111 +- 0.1		120
109 +- 0.1	115 +- 0.2		123
115 +- 0.1	122 +- 0.1		128
120 +- 0.1	126 +- 0.1		133
130 +- 0.1	136 +- 0.1		143

Table S2: Temperature protocol for samples A and B, as well as the external calibration measurement. Data shown in the manuscript are shaded yellow. Some lower temperatures are not shown because the dynamics in the steps in between do not show significant changes.

Detector images

As the X-ray beam penetrated through the two diamond anvils, Pseudo-Kossel-lines were observed on the detector.[7] The image in Fig. S3C shows a strong line visible at a pressure of 0.75 GPa in the 2D SAXS pattern. These lines do not vanish when rotating the DAC inside the X-ray beam. Weak Pseudo-Kossel lines can also be observed in the 2D WAXS pattern (Fig. 1A), but do not affect the WAXS analysis. The XPCS analysis, however, is done from the SAXS images, and at 0.75 GPa those lines affect the calculated correlation function, even after masking the relevant pixels. Sample D could therefore not be analyzed in terms of dynamics. For lower pressures as in samples A and B, weak Pseudo-Kossel lines can be masked out, without influencing the result. No effect of directionality has been found. Figure S6 shows the masked region on the detector, and g2 curves calculated independently from the upper and lower part of the detector.

For the here described first experiments, we used type Ia diamonds. Later experiments indicated that this effect can be reduced by the use of type IIa diamonds.

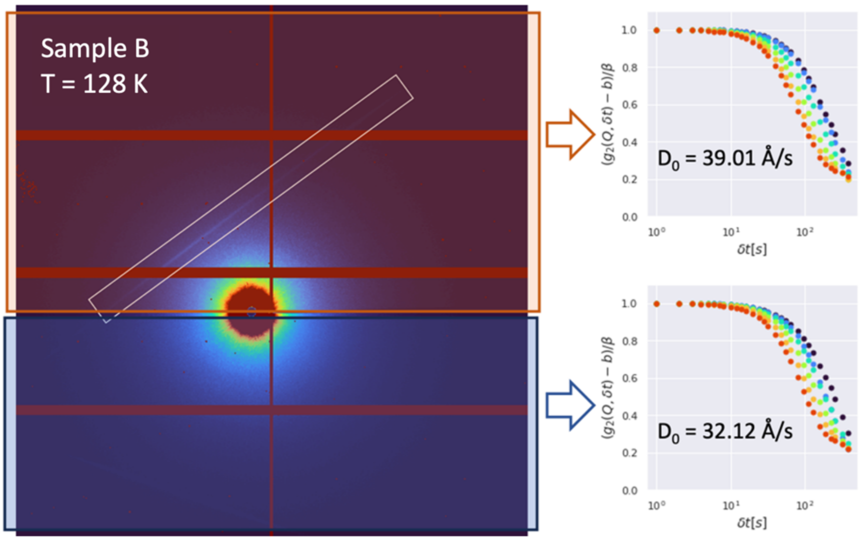

Error! Bookmark not defined.Fig. S6: Example for g_2 curves of sample B at 128 K calculated separately from the upper and lower part of the detector.

Sample A

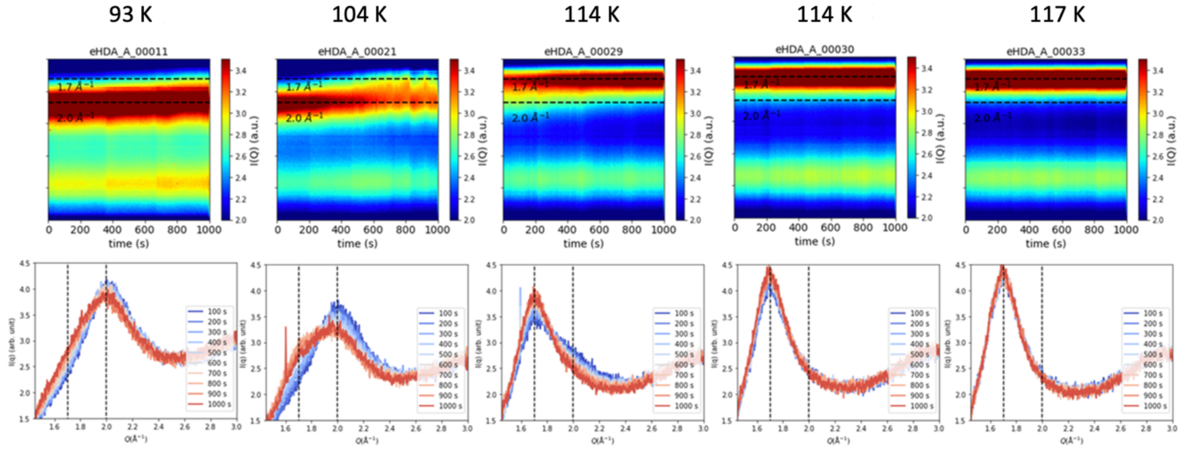

Fig. S7: Wide angle X-ray scattering intensity. The time evolution of the first diffraction maximum for sample A is plotted.

Figure S8, top row, shows the g_2 functions and the related fits at different Q. In the bottom row, the fit results for the Kohlrausch–Williams–Watts (KWW) exponent γ are shown. This is related to Fig. 2 in the main manuscript.
In Fig 3 of the main manuscript the two-time correlation functions (TTC) of the first 300 s of experimental time are displayed. In total we measured 1000 scattering patterns, each pattern was exposed to X-rays for 1s. The TTCs for the complete 1000 s are shown in Fig. S9.

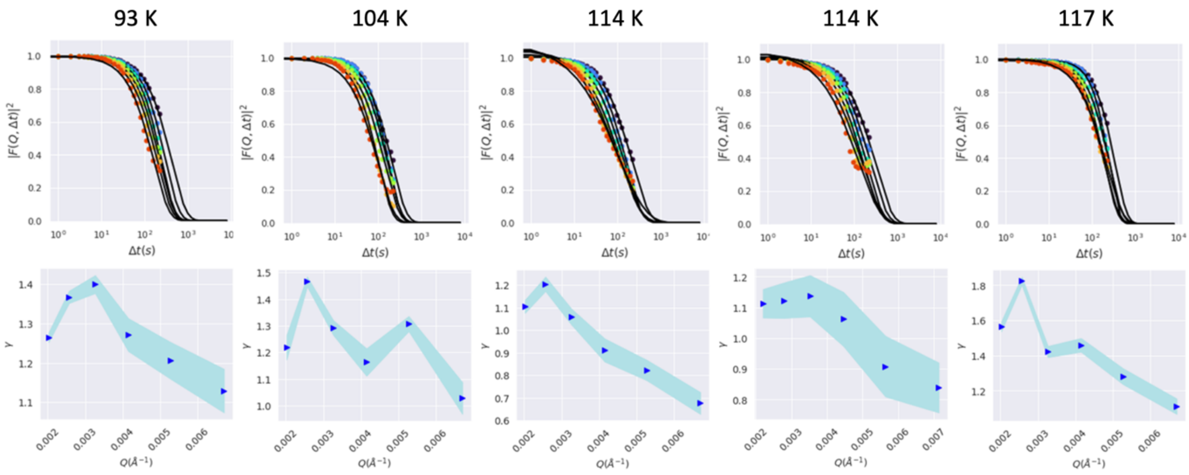

Fig. S8: (Top row) Autocorrelation functions g_2 and the corresponding Kohlrausch–Williams–Watts (KWW) fits for sample A. (Bottom row) Fit results for the KWW exponent γ.

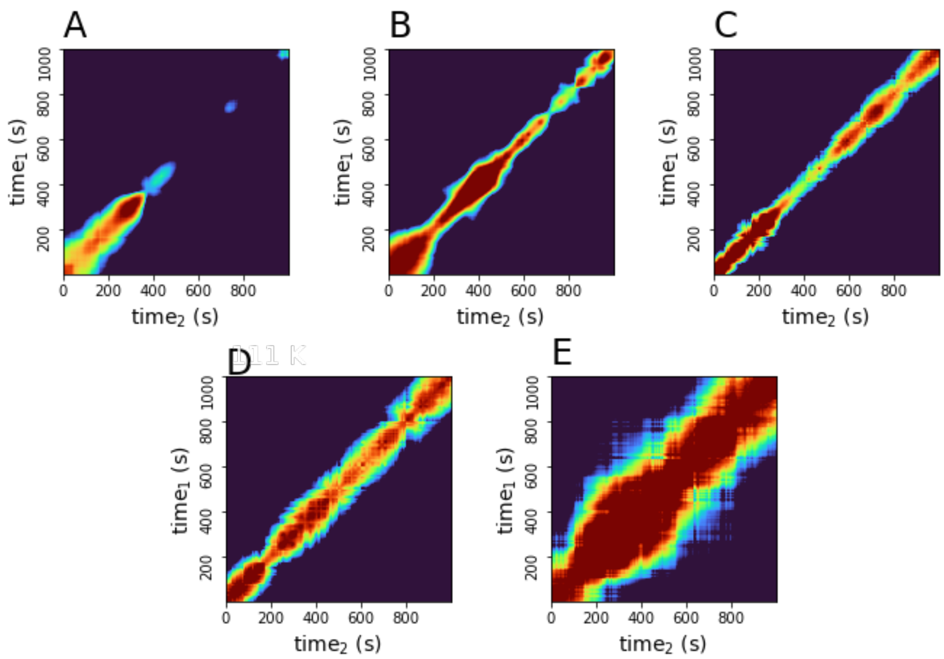

Fig. S9: Two-time correlation functions for sample A at Q = 0.0033 $Å^{-1}$ and at temperatures T_{sample} = 93 K (A), 104 K (B), two times 114 K (C, D) at two different sample positions, and 117 K (E).

Sample B

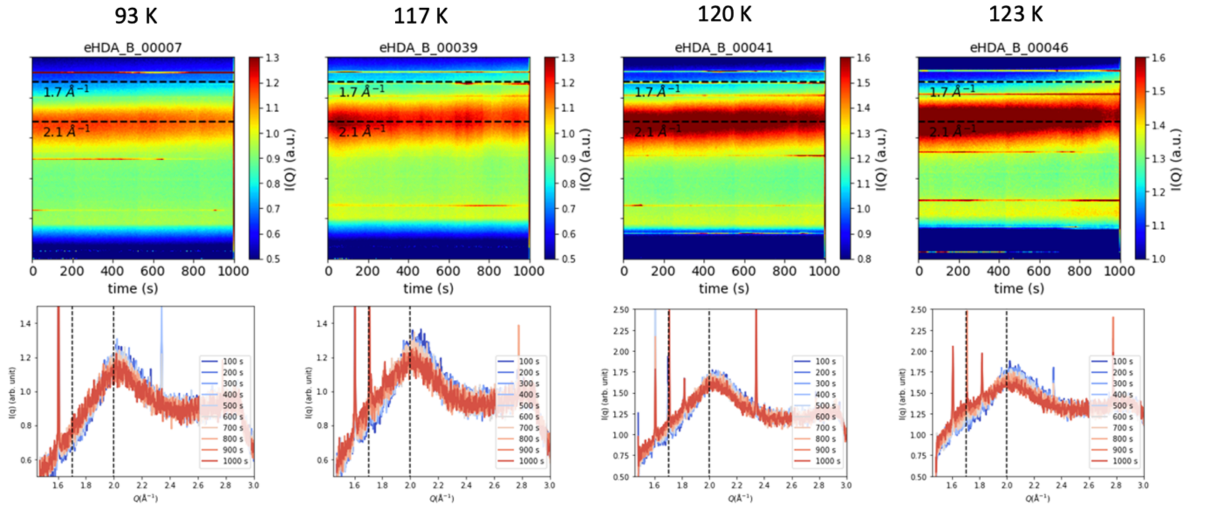

Fig. S10: Wide angle X-ray scattering intensity. Time evolution of the first diffraction maximum.

In the main manuscript we discussed that the correlation functions of sample B (Fig. 5) can be best modelled by simultaneously fitting two dynamical components (see Fig. 6). We additionally tested a single exponential KWW decay fit using Equation (3) of the main manuscript, the results are shown in Fig. S11. The middle row shows the corresponding KWW-exponent (γ) of the fit. The Q-dependencies of the relaxation time (τ) are shown in the lower row. It is evident from this figure that a single (diffusive) component cannot fit the data, as the relaxation times do not display a clear Q^2-dependence, the fit rather exhibits a clear offset. Therefore, a multicomponent fit was applied in the main manuscript.

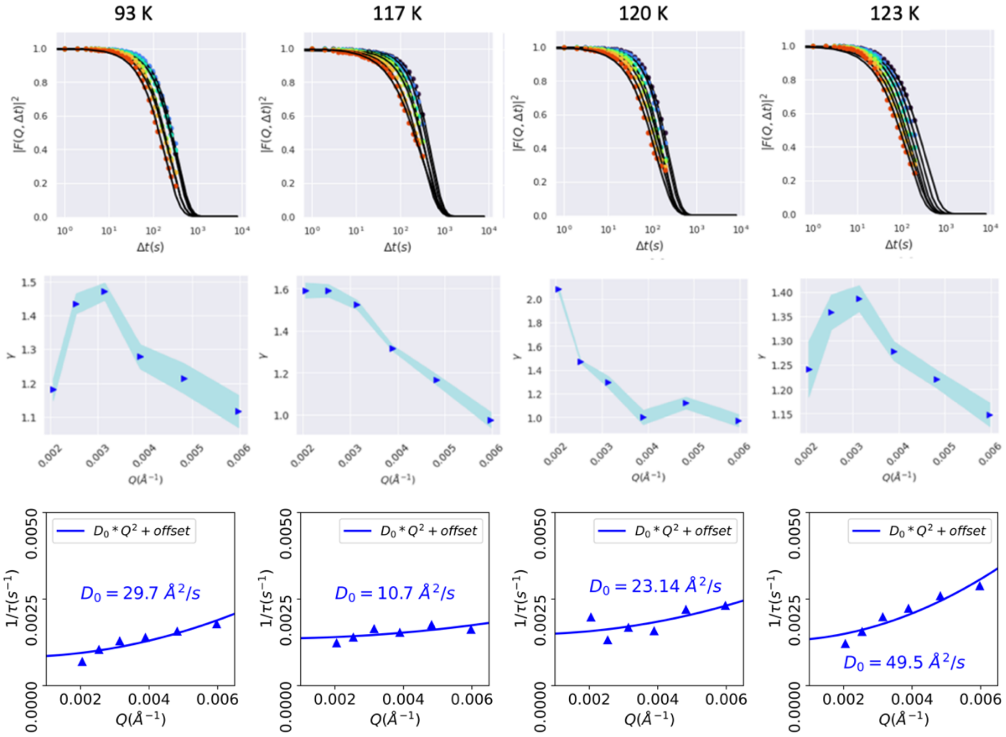

Fig. S11: (Top row) Autocorrelation functions g_2 and the corresponding KWW fits of sample B, calculated for the first 500 s of a series at Q = 0.0035 $\AA^{-1}$. (Middle row) The results for the KWW exponent of fitting the g_2 functions of sample B. (Lower row) Q-dependency of the relaxation time τ.

Figure S12 in addition shows the XPCS analysis after the transition to LDL or LDA and ice I. The question at hand is, which of the processes dominates, as it is very difficult to distinguish. A separate measurement on pure LDA over the whole temperature range would be necessary to confirm a potential liquid-liquid transition free of doubt.

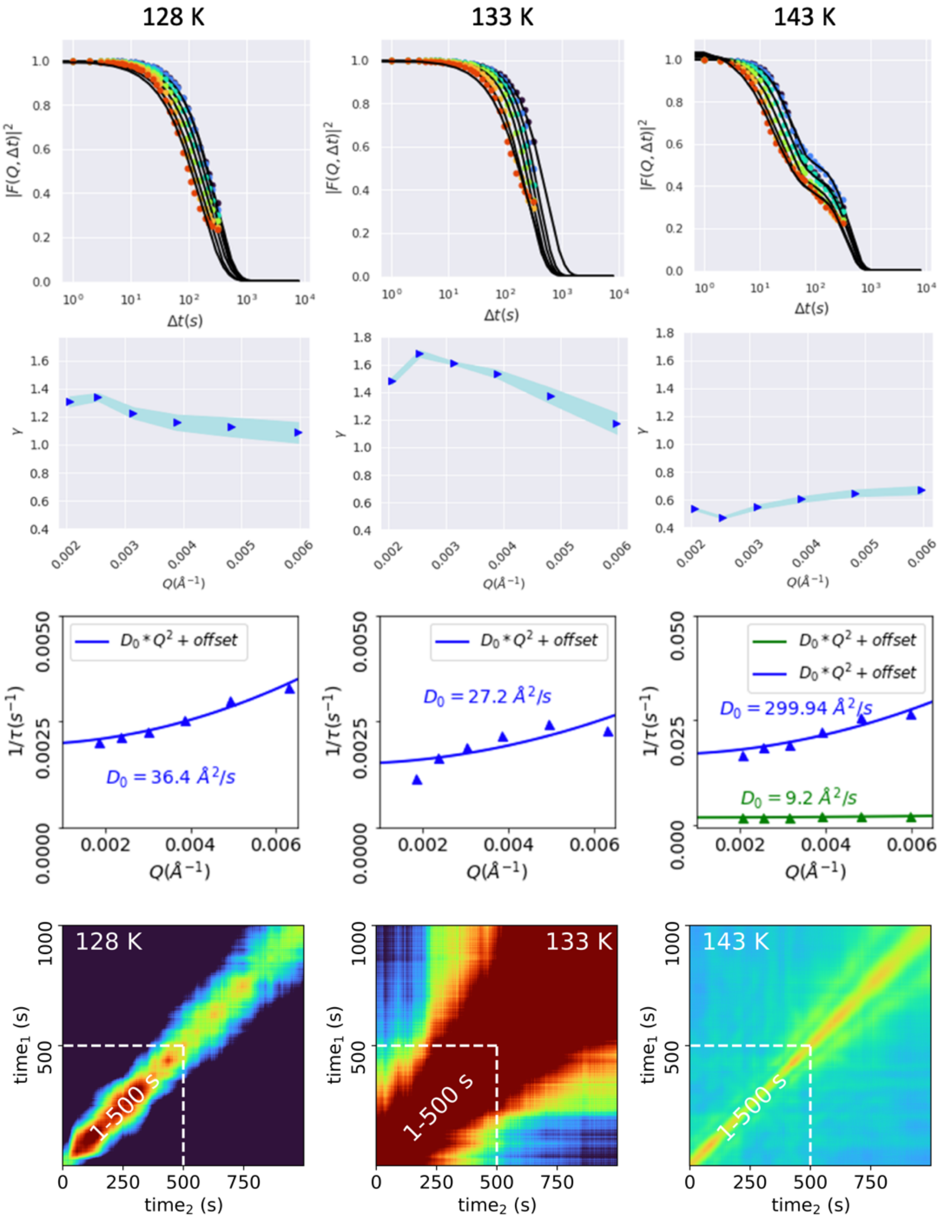

Fig. S12: (Top row) Autocorrelation functions g_2 and the corresponding KWW fits of sample B, calculated for the first 500 s of a series at Q = 0.0035 $Å^{-1}$. (Third row) The results for the KWW exponent of fitting the g_2 functions of sample B. (Third row) Q-dependency of the relaxation time τ. (Lower row) TTCs of the three measurements.

At 128 K and 133 K the data let us hints towards a liquid-like character of the sample (LDL), similar to the previous temperature, based on the fits shown in Fig. S11. The dataset right after the transition shows a Q^2-dependence and a diffusion coefficient of D = 36.4 $Å^2$/s. The Kohlrausch–Williams–Watts (KWW) exponent γ of the correlation decay approaches 1, which hints towards Brownian motion. Still, the Q-dependency of the relaxation time τ exhibits an offset, and therefore no fully Brownian motion is observed. At higher temperatures a deceleration can be observed in the TTCs, this behavior is clearly opposite to the transition

from high-to low-density. At 143 K this is accompanied by a second dynamical process, and a pronounced double exponential decay is visible, both in the g2 curves as well as in the TTC. In addition, γ for the faster process clearly changes to be < 1. The WAXS I(Q) at this temperature shows a small amount of crystalline ice, but no drastic growth. Therefore, the observed dynamics can be related to both, formation of LDL as well as crystalline ice.

Simultaneously fitting of g2 curves with two dynamical components

In the main manuscript we discussed that the correlation functions of sample B (Fig. 5) can be best modelled by simultaneously fitting two dynamical components (see Fig. 6). The model in equation (5) is a linear combination of a ballistic and a diffusive component, with v as velocity and D as diffusion coefficient:

$$g_2(Q,\Delta t) = b(Q) + \beta(Q) \cdot \left(Ae^{-(vQ\Delta t)^2} + (1-A)e^{-(DQ^2\Delta t)^\gamma}\right)^2.$$

The parameters are as follows: baselines $b(Q)$, contrasts $\beta(Q)$, ballistic amplitude A, diffusive KWW exponent γ, ballistic velocity v, and diffusivity D. The model is applied to all data points $g_2(Q,\Delta t)$ in a measurement series with a least-squares surface fit. For optimization, we used the *curve_fit* function from SciPy's *optimize* subpackage [8] (with the default trust region reflective minimization algorithm). There is one baseline parameter b and one contrast parameter β per Q-value, so in total, $2{*}N_Q{+}5$ parameters are fitted to each measurement (N_Q is the number of Q bins).
We used a calibration measurement of an Aerogel sample to establish the maximum contrast, here determined to be ~9%. Baselines which are affected by e.g. streaks or a Kossel-line, given by the difference of 1 and the g_2 value at $t \to 0$, are corrected by the aerogel contrast. The fit results for contrast parameter β and baseline parameter b, related to Fig. 6 of the main manuscript, are shown in Fig. S13. The contrast drop with increasing momentum transfer Q matches our expectations.

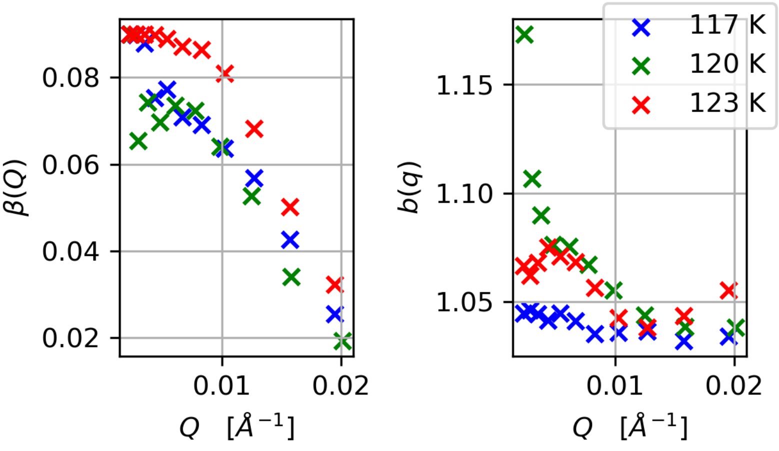

Fig. S13: Contrast β and baseline values b determined by fitting to the autocorrelation functions g_2 of sample B. This figure relates to Fig. 6 of the main manuscript, values shown for temperatures T_{sample} = 117 K, 120 K, 123 K.

1. Winkel, K., et al., *Water Polyamorphism: Reversibility and (Dis)continuity.* Journal of Chemical Physics, 2008. **128**(4): p. 6.
2. Mariedahl, D., et al., *X-ray Scattering and O–O Pair-Distribution Functions of Amorphous Ices.* The Journal of Physical Chemistry B, 2018. **122**(30): p. 7616-7624.
3. Seidl, M., et al., *From Parallel to Single Crystallization Kinetics in High-Density Amorphous Ice.* Physical Review B, 2013. **88**(17): p. 6.
4. Seidl, M., et al., *Shrinking water's no man's land by lifting its low-temperature boundary.* Physical Review B, 2015. **91**(14): p. 14.
5. Nelmes, R.J., et al., *Annealed High-Density Amorphous Ice Under Pressure.* Nature Physics, 2006. **2**(6): p. 414-418.
6. Loerting, T., et al., *Cryoflotation: Densities of Amorphous and Crystalline Ices.* Journal of Physical Chemistry B, 2011. **115**(48): p. 14167-14175.
7. Okada, M. and H. Iwasaki, *X-ray diamond anvil cell and Pseudo-Kossel line pattern.* physica status solidi (a), 1980. **58**(2): p. 623-628.
8. Virtanen, P., et al. *SciPy 1.0: fundamental algorithms for scientific computing in Python*. Nat Methods **17**, 2020P: p.261-272. https://doi.org/10.1038/s41592-019-0686-2
